# A case of esophageal cancer in a diverticulum treated by surgical resection: a case report

**DOI:** 10.1186/s44215-023-00123-5

**Published:** 2023-11-20

**Authors:** Momoko Fukuda, Toru Aoyama, Norio Yukawa, Mihwa Ju, Kazuki Kano, Tetsushi Ishiguro, Keisuke Kazama, Sho Sawazaki, Hiroshi Tamagawa, Ryosuke Ikeda, Hiroaki Kaneko, Shin Maeda, Aya Saito, Yasushi Rino

**Affiliations:** 1 Department of Surgery, Yokohama City University School of Medicine, 3-9 Fukuura Kanazawa-ku, Yokohama City, Kanagawa Japan; 2 Department of Gastroenterology, Yokohama City University School of Medicine, 3-9 Fukuura Kanazawa-ku, Yokohama, Kanagawa Japan

**Keywords:** Esophageal diverticulum, Esophageal cancer in diverticulum, Esophageal cancer, Zenker’s diverticulum

## Abstract

**Background:**

An esophageal diverticulum is a relatively rare disease, with reports of treatment for esophageal cancer in the diverticulum even rarer.

**Case presentation:**

The case involved a 72-year-old male with a chief complaint of dysphagia. He was diagnosed with an esophageal diverticulum (Zenker’s diverticulum) measuring 10 cm in diameter. Five years later, an upper gastrointestinal endoscopy revealed an iodine-unstained 0–IIb lesion of 20 mm in diameter with type B1 vessels in the diverticulum. An endoscopic biopsy and CT revealed it to be squamous cell carcinoma, cT1a-EP/LPM N0 M0, cStage 0. Because the lesion was in the diverticulum and endoscopic resection was difficult with the risk of perforation, surgical resection was set as the course of treatment. Diverticulectomy was performed via a cervical approach, using a stapler, and the patient was discharged on the 16th day without any complications. The pathological diagnosis was pTis-EP, ly0, v0, R0.

**Conclusions:**

We think this case is very rare and diverticulectomy of early esophageal cancer in the diverticulum is available and safe.

## Background

The esophageal diverticulum is a relatively rare disease; furthermore, there are few reports on esophageal cancer in the diverticulum, with subtotal esophagectomy and lymphadenectomy proactively performed as treatments. We report the case of a 72-year-old man whose workup supported the diagnosis of esophageal cancer in the diverticulum. On exploration, the lesion was found to be an early esophageal cancer in the diverticulum. The lesion was successfully managed with diverticulectomy using surgical stapling. This work has been reported in line with the SCARE criteria [1].

## Case presentation

**Fig. 1:**
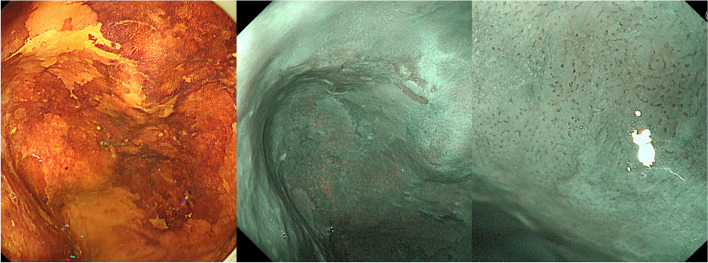
Endoscopic image. Left: an iodine-unstained 0–IIb lesion of 20 mm in diameter in the esophageal diverticulum, center: a photo with narrow-band imaging, right: a photo with image-enhanced endoscopy with magnification

**Fig. 2:**
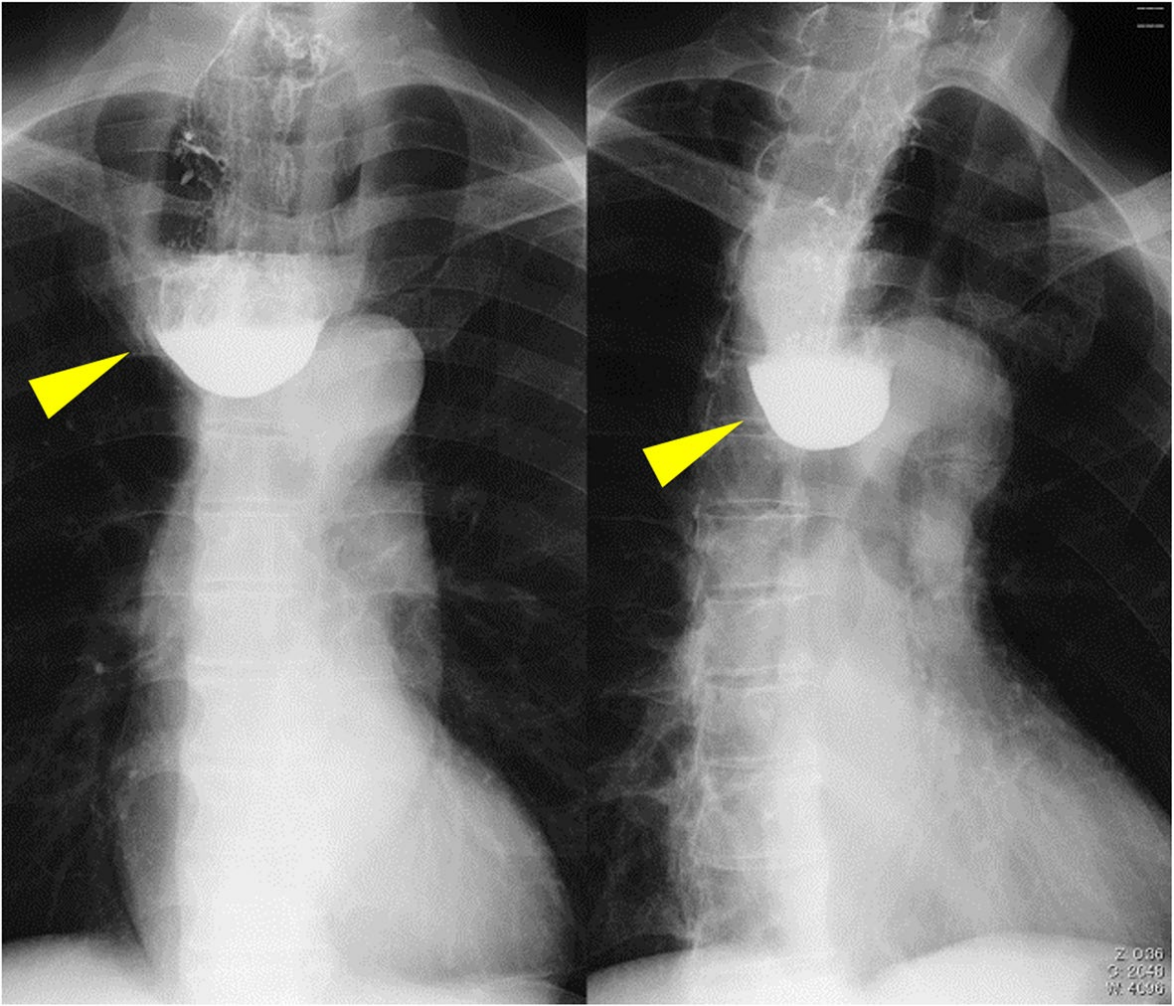
Upper gastrointestinal contrast examination. Left: center, right: first oblique. A Zenker’s diverticulum measuring 10 cm in diameter was found in the cervical esophagus

**Fig. 3:**
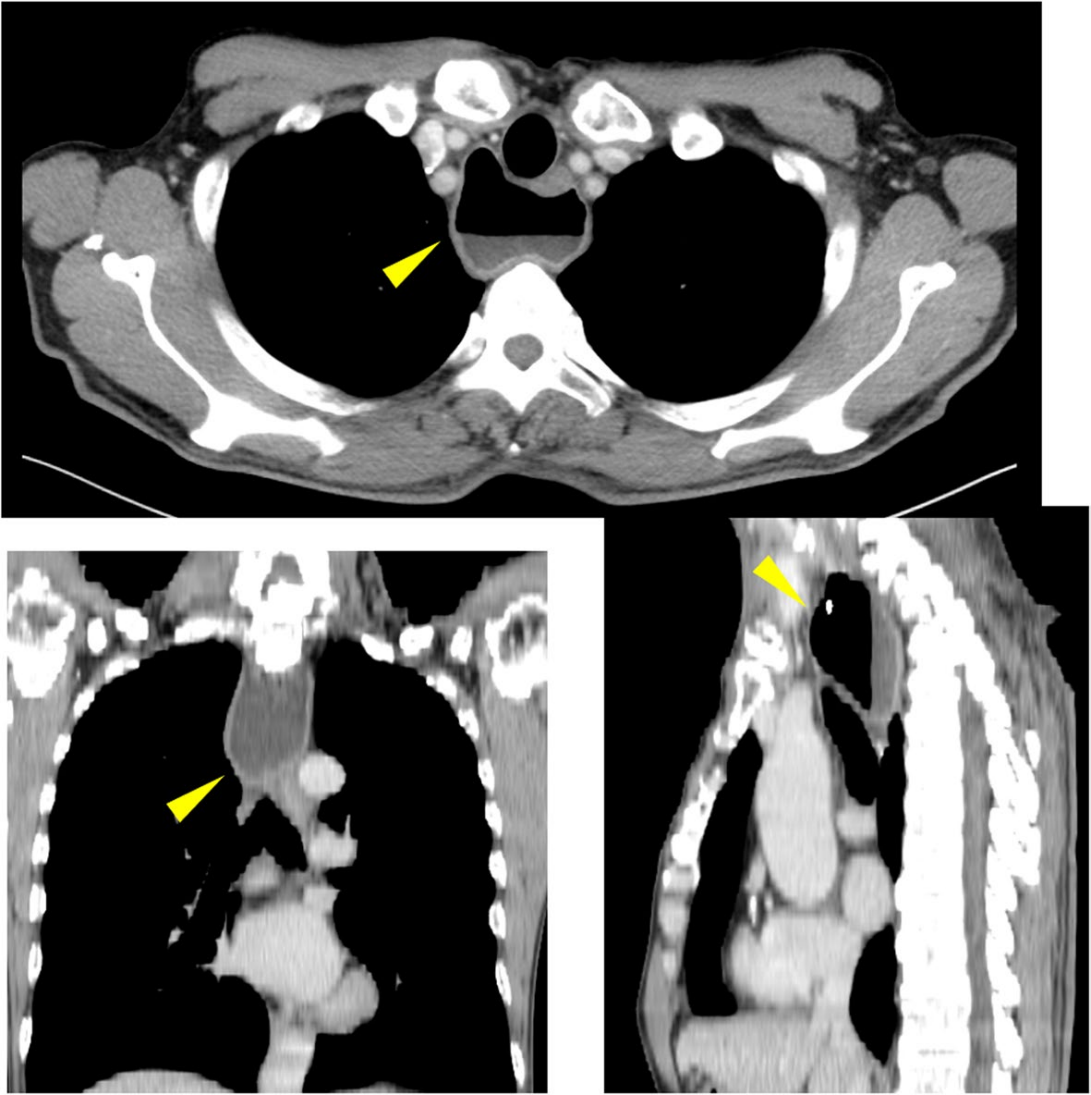
Chest contrast-enhanced CT scan. A diverticulum was observed in the cervical esophagus with fluid retention inside. Tumor localization is unclear

A 72-year-old healthy man was referred to our institution with progressive dysphagia to solids. He reported a recent episode of solid food getting stuck in his throat, which prompted presentation to an outside endoscopist. The patient reported alcohol as 1 cup (180 ml) of sake/day (5 days a week) use. He was a current smoker with a 52-pack-year history. The patient had a past medical history of hypertension. He was diagnosed with an esophageal diverticulum (Zenker’s diverticulum) and reflux esophagitis equivalent to Los Angeles grade A 5 years ago. Subsequently, undergoing outpatient follow-ups were started, at his request. Five years later, gastrointestinal endoscopy revealed an iodine-unstained 0-IIb lesion of 20 mm in diameter with type B1 vessels in the esophageal diverticulum. It was diagnosed as squamous cell carcinoma by biopsy (Fig. 1). Esophagography demonstrated that Zenker’s diverticulum measuring 10 cm in size was found at the entrance of the esophagus on the left side of the neck. No tumor was observed in the diverticulum (Fig. 2). Computed tomography (CT) demonstrated a 10-cm esophageal diverticulum on the left side of the neck, but the localization of the tumor was unclear. No lymphadenopathy and no metastasis to any other organs were observed (Fig. 3).

Finally, we diagnosed esophageal cancer cT1a-EP/LPM, N0, M0, cStage 0. Physical exam and laboratory testing were unremarkable. Although it was an early-stage esophageal cancer, endoscopic treatment was considered inappropriate due to the risk of perforation. Therefore, surgical treatment was chosen.

**Fig. 4:**
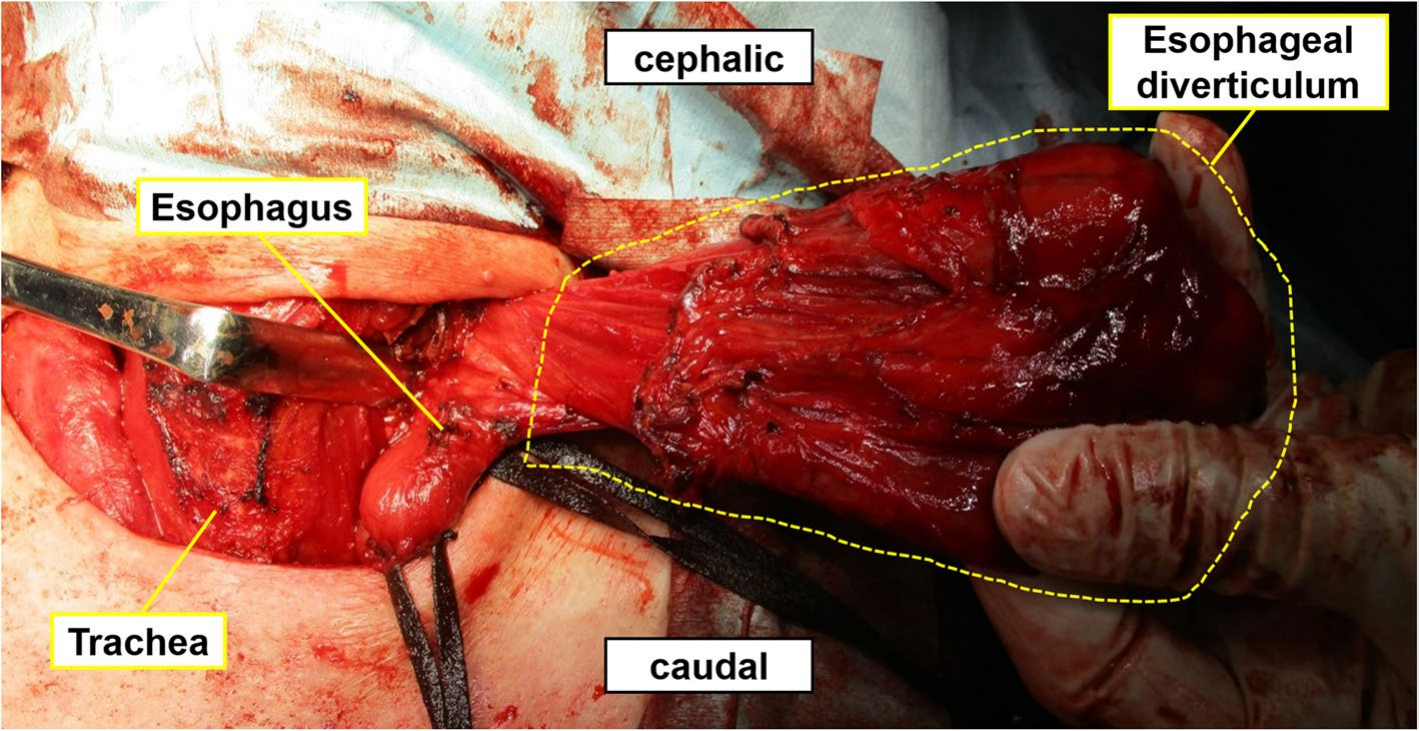
Intraoperative findings. View of the esophageal diverticulum pulled out of the body from the neck.

The surgery began via a left cervical approach through collar incisions of 6 cm in width. The esophagus was dissected from the trachea in the neck region, and the adhesions of the diverticulum on the dorsal side of the esophagus were dissected in order to pull out the diverticulum. Complete mobilization revealed that the stalk was identified and the diverticulum could be delivered to the neck incision. Repeat endoscopy during the surgery demonstrated an ostium in the esophageal wall opening into a blind-ending pouch and a cancer lesion. The diverticulum including the cancer lesion was fully mobilized and resected using a stapler completely (Fig. 4). Mucosal closure was reinforced with overlying muscle. No lymph nodes swelling and no invading cancer to the cut-end were observed macroscopically. There was no stenosis or leakage. His oral intake was good, and he was discharged on the 16th postoperative day.

**Fig. 5:**
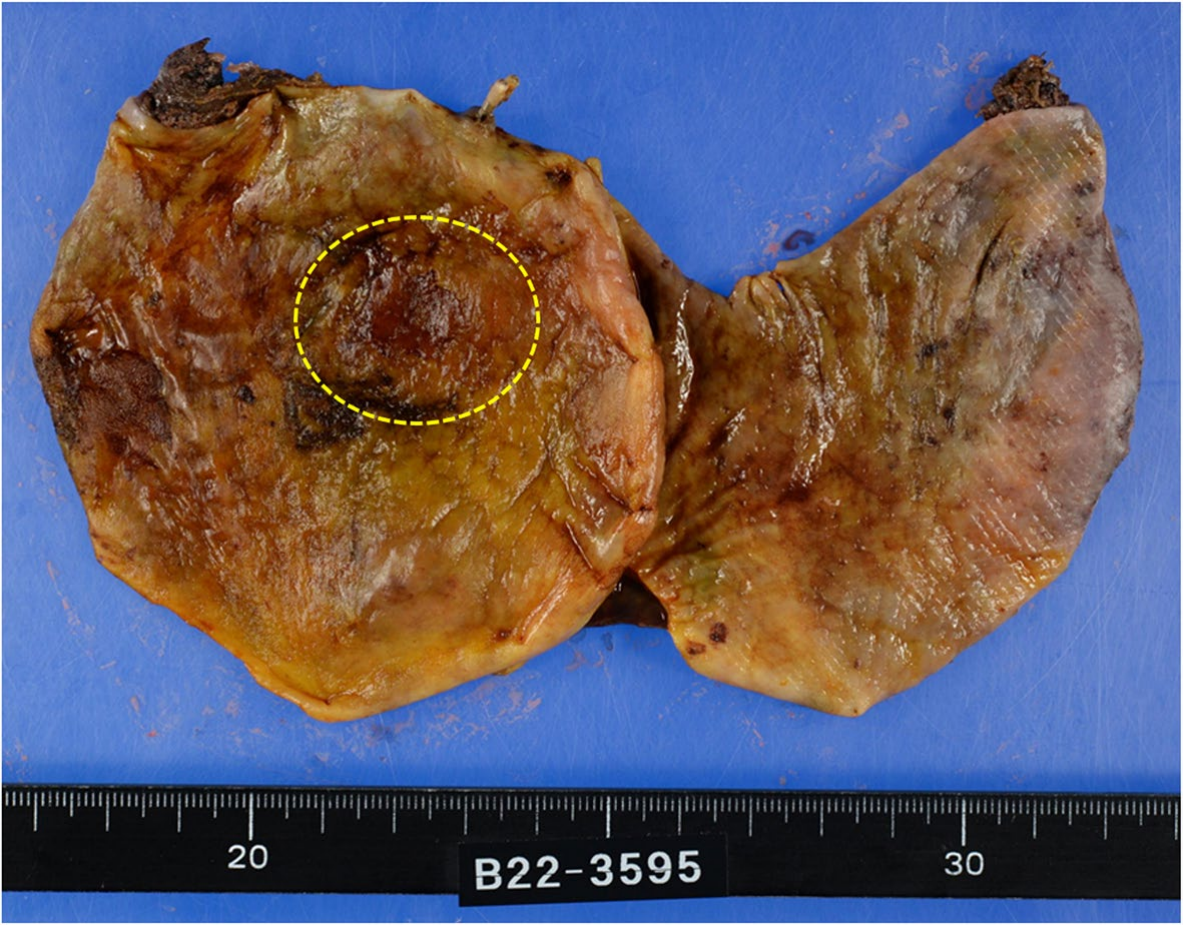
Resection specimen (findings). A resected and expanded esophageal diverticulum. Mucosal surface after iodine application. A tumor is shown within the yellow dotted line

The resected specimen was a cystic esophageal wall, and 0–IIb lesions measuring 20×5 mm, showing non-staining of iodine, were macroscopically observed on the mucosal surface. Histologically, atypical cells with hyperstaining chromatin, large and small immobile swollen nuclei, proliferated throughout the epithelium, which was consistent with the lesion, were p53 diffusely positive and Ki-67-positive, indicating squamous cell carcinoma. The tumor was confined to the epithelium with no obvious stromal invasion (pTis-EP). The histological tumor diameter was 17×7 mm, no lymphatic or venous invasion was observed, and the resection margins were negative (pN0, lu0, v0, R0). The cyst wall was formed from the esophageal mucosa to the submucosa and was a pseudodiverticulum without the muscularis propria (Fig. 5).

## Discussion

Esophageal diverticulum is a relatively rare disease with a prevalence of 0.06 to 3.6% based on radiologic and endoscopic examinations [2, 3]. In Japan, some Japanese authors reported an incidence of approximately 1% among all gastrointestinal diverticula and more commonly found in males in their 50s to 70s [4, 5]. According to reports made in Japan, the common sites of esophageal diverticulum include three sites: *pharyngeal esophageal* diverticulum (Zenker’s diverticulum) in 15.8%, tracheal bifurcation diverticulum (Rokitansky diverticulum) in 73.0%, and supradiaphragmatic diverticulum in 11.2% [6]. The common sites in other countries have been indicated as 54.7%, 26.2%, and 12.7%, respectively [7]. Zenker’s diverticulum is relatively rare in Japan. We performed a literature review of esophageal diverticula and cancer. The incidence of cancer in a diverticulum is 0.3–7, 1.8, and 0.6% for pharyngoesophageal, midesophageal, and epiphrenic diverticula, respectively [8].

In Japan, esophageal cancer in the diverticulum coexists in 0.6% of esophageal diverticula, and like other esophageal cancers, elderly men are more likely to be affected [5]. Carcinogenesis in a diverticulum is thought to be caused by chronic irritation and inflammation due to food stasis [9–11]. Furthermore, because cancer in pseudodiverticula lacks the muscularis propria compared to a true diverticulum, it is believed that it reaches the adventitia early and tends to infiltrate the surrounding organs [12]. Surgical treatment is selected for symptomatic cases or cancer in the diverticulum, with surgical methods for esophageal diverticulum including diverticulopexy, diverticulectomy, and pharyngeal myotomy. However, patients with malignant tumor complications may find that the depth of cancer invasion is deeper than expected due to the structure of the diverticulum, so esophagectomy and lymphadenectomy are proactively performed due to many reports of advanced cancer [13, 14]. In addition, the wall is often found to be thin due to the loss or tearing of the muscular layer of the diverticulum wall, including cases of perforation at the time of treatment; therefore, endoscopic treatment for cancer in the diverticulum should be carefully considered [12, 14]. Although squamous cell carcinoma is the most common histological type of cancer in the diverticulum, one case of adenocarcinoma has been reported, suggesting that it originates from the ectopic gastric mucosa [15].

Although endoscopic treatment for a T1a-EP/LPM lesion would be selected, due to the occurrence of the lesion in the diverticulum, surgical treatment was performed in this case, considering the risk of perforation. While lymph node metastasis is rare in EP/LPM cancer and lymph node dissection can generally be omitted, in this case as well, it was confirmed that there was macroscopically no peripheral lymphadenopathy during the operation, so diverticular resection was performed without lymphadenectomy. The esophageal diverticulum may be asymptomatic and the incidence of esophageal cancer in diverticula is extremely low; however, if it develops in a diverticulum that is larger than a certain size, it is unlikely that obstruction will occur because the esophageal lumen is maintained even if it progresses. Therefore, it is most commonly detected as advanced cancer, the prognosis for which is considered poor [16]. It is important to be aware of the need for diagnosis and follow-up among patients in which a diverticulum has already been pointed out.

Intraoperatively, we were able to identify the bilateral recurrent and vagus nerves and stimulated them with NIM multiple times, and checked the nerve response to avoid paralysis. Generally, it has been reported that recurrent nerve palsy after esophageal cancer surgery is about 49.2% immediately after surgery and 18.6% in the first 6 months after surgery [17]. But in this case, as of 1 year after surgery, no symptoms such as hoarseness or dyspnea were observed, and it was thought that the patient was not suffering from postoperative recurrent nerve palsy. The diverticulum was resected using a stapler parallel to the long axis of the esophagus. To prevent postoperative suture stenosis, an intraoperative endoscope was inserted into the esophagus caudal to the diverticulum, which was used as a stent while resecting the diverticulum. As of 1 year postoperatively, the patient has had no dyspnea or dysphagia.

Japanese 29 reports on esophageal cancer in diverticula undergoing surgery, similar to this case, were found in *ICHUSHI* medical journals when searching using keywords such as “esophageal cancer in the diverticulum” and “surgery” (Table 1). Patients ranged in age from 34 to 78 (average 64.0), with an average age of 66.6 for males and 58.9 for females, suggesting that males tended to be older. The male-to-female ratio was 19:10, with 65.5% male and 34.5% female, indicating it was more common in males as previously reported. The majority of cases included: 2 cases of cervical esophagus Ce (8.7%), 2 cases of upper thoracic esophagus Ut (8.7%), 10 cases of upper thoracic esophagus Mt (43.5%), 8 cases of lower thoracic esophagus Lt (34.8%), 1 case abdominal esophageal Ae (4.3%), and many Mt and Lt cases, with only 2 cases of Ce, which is the main locus of Zenker’s diverticulum, similar to this case. The degree of tumor progression indicated Stage 0 in 7 cases (35%), Stage I in 5 cases (25%), Stage II in 3 cases (15%), Stage III in 1 case (5%), and Stage IVa in 4 cases (20%), many of which were found in advanced cancers. The treatment of esophageal cancer in the diverticulum included esophagectomy in 16 cases (59.3%), diverticulectomy in 5 cases (18.5%), endoscopic treatment in 2 cases (7.4%), radiation therapy in 2 cases (7.4%), and chemoradiotherapy in 1 case (3.7%). The average prognosis was 40.0 months for all known cases and 42.4 months for those who survived without recurrence. Recurrence and death occurred in 3 cases, occurring after 3.5 months (death due to other diseases), 6 months (death due to local recurrence/pulmonary metastasis), and 56 months (local recurrence/cervical lymph node metastasis). Because of these reports, it is important for good prognosis to follow-up among patients in which a diverticulum has already been pointed out.

**Table 1:** Esophageal cancers in diverticula reported in Japan [18–35]

	Year	Age	Sex	Chief complaint	Lesion	Type	Histological type	Depth of invasion	Treatment	RFS (duration of observation: the event)
1	1982	67	M	Epigastric pain	Mt	0-IIa+IIc	SCC	T1a	Subtotal esophagectomy	7 months (7 months: recurrence-free)
2	1988	47	F	Dysphagia	Ut	-	SCC	-	Radiation	5 years (5 years: recurrence-free)
3	1990	68	M	Chest pain	Mt	-	SCC	T1b	NAC and subtotal esophagectomy	3.5 months (3.5 months: death for other disease)
4	1996	54	F	Asymptomatic	-	0-IIa	SCC	T1a	Esophagectomy	50 months (50 months: recurrence-free)
5	1996	74	F	Asymptomatic	Lt	0-IIc	SCC	T1a	Lower third of esophagus resection and proximal gastrectomy	11 months (11 months: recurrence-free)
6	2000	62	M	Asymptomatic	Mt	-	-	-	Diverticulectomy	56 months (56 months: recurrence-free)
7	2003	57	M	Anorexia, weight loss	Ut	1	SCC	T2	Subtotal esophagectomy	14 months (14 months: recurrence-free)
8	2004	75	M	Chest pain	Mt	0-IIa	SCC	T1b	Subtotal esophagectomy	21 months (21 months: recurrence-free)
9	2004	64	M	Chest pain	Mt	0-IIc	SCC	T1b	NAC and subtotal esophagectomy	1 year (1 year: recurrence-free)
10	2007	71	M	-	Mt	0-IIa	-	T1a	Radiation	5 years (5 years: local recurrence)
11	2007	78	M	-	Ce	0-IIa+IIb	SCC	T1a	Diverticulectomy, radiation	56 months (56 months: local recurrence)
12	2007	67	M	Chest discomfort	Mt-Lt	0-IIa	SCC	T1b	Subtotal esophagectomy	31 months (31 months: recurrence-free)
13	2007	54	F	-	Mt	0-IIc	SCC	T1a	Subtotal esophagectomy	14 years (14 years: recurrence-free)
14	2007	74	F	-	Lt	0-IIa	SCC	T1a	Subtotal esophagectomy	11 years (11 years: recurrence-free)
15	2007	58	M	Vomiting	-	-	-	T1a	Diverticulectomy	5 years (5 years: recurrence-free)
16	2007	70	M	Dysphagia, Weight loss	Lt	-	SCC	T4a	Surgery	-
17	2007	34	F	Heartburn, dysphagia	Lt	-	SCC	-	Surgery	-
18	2009	78	F	Vomiting, cough	Mt	3	SCC	T4c	Chemoradiotherapy	5 months (5 months: local recurrence, 6 months: death)
19	2010	69	M	Epigastric pain	Mt	-	SCC	T2	Subtotal esophagectomy	4 years (4 years: recurrence-free)
20	2012	36	M	Asymptomatic	Lt	-	GIST	-	Diverticulectomy	19 months (19 months: recurrence-free)
21	2012	62	F	Chest discomfort	Ae	-	-	T3	Laparoscopic surgery	-
22	2013	57	F	Asymptomatic	Ce	0-IIb	-	T1a	Diverticulectomy	-
23	2013	72	M	Asymptomatic	-	0-IIc	SCC	T1a	Esophagectomy	6 months (6 months: recurrence-free)
24	2014	55	F	Pneumonia	Lt	3	SCC	T4b	Esophagectomy, right lower lobectomy	-
25	2017	75	M	Chest pain, melena	-	1	SCC	T3	NAC and subtotal esophagectomy	13 months (13 months: recurrence-free)
26	2019	73	M	-	Mt	-	SCC	T1a	ESD	-
27	2019	73	M	Asymptomatic	-	0-IIb	SCC	T1a	ESD	-
28	2019	68	M	Vomiting, weight loss	Lt	3	SCC	T4a	Esophagectomy, left lower lobectomy, hepatic left lateral segmentectomy, partial left diaphragm resection	1 year (1 year: recurrence-free)
29	2020	63	M	Asymptomatic	Lt	-	SCC	T3	Esophagectomy	3 years (3 years: recurrence-free)

*RFS* relapse-free survival, *SCC* squamous cell carcinoma, *NAC* neoadjuvant chemotherapy, *GIST* gastrointestinal stromal tumor, *ESD* endoscopic submucosal dissection

In conclusion, we experienced a case in which early-stage esophageal cancer in the diverticulum was discovered in a patient undergoing regular observation of the esophageal diverticulum and in which minimally invasive surgical resection was successful. It is more likely to progress without symptoms when carcinoma develops, compared to normal esophageal cancer. In cases with a diagnosis of esophageal diverticulum, it is possible to make a diagnosis of early esophageal cancer with strict follow-up. We think that diverticulectomy could be available and safe for early esophageal cancer.

## Data Availability

Data sharing is not applicable to this article as no datasets were generated or analysed during the current study.
